# Biogenic amines in the testis: sources, receptors and actions

**DOI:** 10.3389/fendo.2024.1392917

**Published:** 2024-06-20

**Authors:** Monica Beatriz Frungieri, Artur Mayerhofer

**Affiliations:** ^1^ Laboratorio de neuro-inmuno-endocrinología testicular, Instituto de Biología y Medicina Experimental (IBYME), Fundación IBYME, Consejo Nacional de Investigaciones Científicas y Técnicas (CONICET), Ciudad de Buenos Aires, Argentina; ^2^ Biomedical Center Munich (BMC), Cell Biology, Anatomy III, Faculty of Medicine, Ludwig Maximilian University of Munich, Planegg-Martinsried, Germany

**Keywords:** catecholamines, serotonin, histamine, melatonin, testis, male infertility, aging

## Abstract

Biogenic amines are signaling molecules with multiple roles in the central nervous system and in peripheral organs, including the gonads. A series of studies indicated that these molecules, their biosynthetic enzymes and their receptors are present in the testis and that they are involved in the regulation of male reproductive physiology and/or pathology. This mini-review aims to summarize the current knowledge in this field and to pinpoint existing research gaps. We suggest that the widespread clinical use of pharmacological agonists/antagonists of these signaling molecules, calls for new investigations in this area. They are necessary to evaluate the relevance of biogenic amines for human male fertility and infertility, as well as the potential value of at least one of them as an anti-aging compound in the testis.

## 1 Introduction

Biogenic amines are best known for their roles as neurotransmitters, namely three catecholamines (dopamine –DA–, norepinephrine –NE–, and epinephrine –Epi–), serotonin (5-HT) and histamine (HA) (1). Furthermore, the biogenic amine melatonin is a neurotransmitter-like compound (2).

Catecholamines in the brain play an important role in the secretion of GnRH and the regulation of the pituitary–testicular axis mainly via α1-adrenergic receptors (3–5). The existence of a descending multi-synaptic, pituitary-independent neural pathway linking the hypothalamus and the testis, which involves, at least in part, activation of β-adrenergic receptors, was reported in the male rat (6). D_1_- and/or D_2_-like DA receptors are expressed in GnRH neurons and thereby participate in the regulation of the endocrine function of the testis (3, 7). GnRH neurons also express the HA receptors H_1_ and H_2_ (5, 8), and synaptic contacts between the serotoninergic system and GnRH neurons were described (9). Pineal-derived melatonin, through its binding to specific sites in the suprachiasmatic nucleus and the pars tuberalis, and via modulation of the kisspeptin (kiss) signaling, influences GnRH and the adenohypophyseal gonadotropins (10–12).

Besides central actions, biogenic amines play roles in peripheral organs including the male gonad. This short review attempts to summarize available insights into sources, receptors, signaling mechanisms, local actions and the clinical relevance of biogenic amines in the testis. We also pinpoint research gaps, concerning their role in male reproduction. We want to apologize to authors, whose work we do not mention here, due the short format of this mini-review.

## 2 Biogenic amines in the testis

### 2.1 Sources of biogenic amines

The main common sources of biogenic amines in peripheral organs are innervation, bloodstream and local production. The testicular sources of the three catecholamines (DA, NE and Epi), 5-HT and HA are described in detail below.

#### 2.1.1 Testicular innervation

Studies revealing the existence of a catecholaminergic and a serotonergic innervation of the testis are summarized in **Table 1**. In human and non-human primates, adrenergic sympathetic innervation of the testis is documented. In addition, intrinsic neuron-like cell bodies with a bipolar or a multipolar elongated phenotype (19–21) were reported, based on the immunodetection of typical markers such as tyrosine hydroxylase (TH). Age-related differences in the number of neuronal elements were found in the testes of the Rhesus monkey. Neuron-like cell bodies were only detected in immature animals. Nerve fibers were observed at all ages but they were more prominent in prepubertal and pubertal monkeys (19, 20). A significant increase in the number of catecholaminergic fibers and neuron-like cells was described in testicular biopsies of patients with idiopathic infertility (21). These changes were accompanied by increased local immune cells (mast cells and macrophages), possibly hinting to mutual interactions (20, 22, 31, 32). Because in the human testis these immune cells may modulate Sertoli cell activity, androgen production and fibrotic events in the tubular wall (31–35), an interactive network between the testicular catecholaminergic input and the local immune system may exist.

**Table 1:** Catecholaminergic and serotonergic innervation of the testis.

Biogenic Amines	Innervated regions of the testis	Species	References
Catecholamines	Capsule, Mediastinum, Tunica albuginea and vasculosa, Blood vessels near the rete testis, Surrounding the myoid cells of seminiferous tubules. Absent in the parenchyma.	Rodents (i.e. rat, hamster)	(13–18)
	Capsule, Mediastinum, Tunica albuginea and vasculosa, Blood vessels near the rete testis, Surrounding the myoid cells of seminiferous tubules, Interstitium intermingled with Leydig cells, mast cells and macrophages.	Primates (i.e. monkey, human)	(19–27)
Serotonin	Capsule, Interstitium surrounding the Leydig cells.	Rat	(28–30)

Of note, the mentioned studies focus on the catecholaminergic nature of testicular nerve fibers. Yet, in catecholaminergic nerve fibers of the human testis several co-transmitters exist, including peptides such as the neuropeptide Y (NPY), ATP, as well as others (36–38). Once released, these may have important actions, as documented recently for ATP (39, 40). Yet their specific and potentially species-dependent presence in the testis and the interaction with catecholamines are not well known and remain to be fully explored.

#### 2.1.2 Blood circulatory system as a source of testicular biogenic amines

Catecholamines are typical adrenal hormones. In human plasma, resting catecholamine levels range from 30 to 70 pg/ml for Epi, 200 to 300 pg/ml for NE and 25 to 100 pg/ml for DA. Levels increase within minutes upon the onset of stress (41, 42). Specifically, during stress, one can assume that catecholamines can reach the testis in concentrations, which allow interaction with their receptors that were described in different human and rodent somatic cells (22, 43–45).

Blood levels of gastrointestinal tract-derived 5-HT in man range between 200 and 300 ng/ml (42). Via interaction with the serotonergic transporter (SERT) and/or specific testicular receptors described in rodent Leydig, Sertoli and germ cells (spermatogonia and sperm) (43, 46–48), blood-derived 5-HT might also influence testicular processes.

Melatonin, once released from the pineal, is taken up by the testis and modulates testicular function (49–55). In humans, physiological levels of melatonin in the blood range from several pg/ml during the day to more than 50 pg/ml at its nighttime peak (56).

Basal plasma HA levels in humans are normally very low (less than 1 ng/ml) (57) but they significantly rise in anaphylaxis (58). Although the potential impact of blood HA on testicular activity is still controversial, HA receptors and regulatory actions exerted by this biogenic amine were described in the testis (59–61).

#### 2.1.3 Other testicular sources of biogenic amines

Enzymes involved in the biosynthesis of catecholamines and histamine were reported to be expressed in germ cells. Leydig cells express the biosynthetic enzymes of the biogenic amines namely catecholamines, 5-HT, HA and melatonin. Melatonin is also locally produced in peritubular cells of the seminiferous tubules, while testicular immune cells synthesize 5-HT, histamine and melatonin. **Table 2** summaries the pertinent reports.

**Table 2:** Expression of catecholamines, serotonin, histamine and melatonin biosynthetic enzymes in the testis.

Biogenic Amines	Biosynthetic enzymes	Testicular cell type	Species	References
Catecholamines	TH	Leydig cells Germ cells	mouse, human	(62–64)
	AACDC	Leydig cells	human	(63, 64)
	BDH	Leydig cells	human	(63, 64)
Serotonin	TPH	Leydig cells Mast cells	human, hamster	(43, 47, 63)
Histamine	HDC	Leydig cells Germ cells Mast cells Macrophages	mouse, human	(60, 61, 65, 66)
Melatonin	SNAT	Leydig cells Mast cells Peritubular cells	hamster, ram	(52, 54, 67)
	ASMT	Leydig cells Mast cells Peritubular cells	hamster, ram	(52, 54, 67)

TH, tyrosine hydroxylase; AACDC, aromatic amino acid decarboxylase; DBH, dopamine (DA) beta-hydroxylase; PNMT, phenylethanolamine-N-methyltransferase; TPH, tryptophan hydroxylase; HDC, histidine decarboxylase; SNAT, serotonin N-acetyltransferase; ASMT, N-acetylserotonin-O-methyltransferase.

Mast cell store HA and 5-HT in secretory granules, and macrophages express the SERT protein and they have been proposed as an alternative source of HA and 5-HT (68, 69).

Testicular mast cell and macrophage numbers change during sexual development and in idiopathic infertility in monkeys and humans, respectively (19–21). Moreover, alterations in the testicular population of immune cells were observed during sexual maturation, aging as well as in the seasonally breeding Syrian hamster (47, 70). Particularly, the increase in mast cells during sexual development was accompanied by higher local levels of 5-HT and its metabolite 5-hydroxyindoleacetic acid in hamster testes (47).

## 3 Receptors, signaling mechanisms and local actions of biogenic amines in the testis

DA receptors D_1_ and D_2_ were reported in mouse and rat Leydig cells and germ cells (62, 71), and DA was implicated in the regulation of size and proliferation of rat Leydig cells (72). While D_1_ receptors participate in the epigenetic reprogramming, namely histone modifications triggered by cocaine in mice germ cells (73), D_2_ receptors, via decreased cAMP levels, may regulate spermatogenesis and spermiogenesis in rats (74). In addition, DA presumably via D_2_ receptors and AKT/PKB phosphorylation, can modulate sperm capacitation, motility and acrosomal integrity in several species (i.e. rat, stallion, boar) (74–76).

In hamsters, Epi and NE stimulate testicular steroidogenesis via α_1_- and β_1_-adrenergic receptors (43, 77–79). It was reported that α_1_/β_1_-adrenergic receptors interact with local 5-HT_2_ receptors mediating the response of the testicular corticotropin releasing hormone (CRH) system on cAMP and testosterone production (43). Interestingly, the impact of catecholaminergic stimuli on testicular testosterone production in hamsters exposed to a short-day photoperiod is greater than in gonads of animals kept under a long day photoperiod (77, 79).

In α_1b_-adrenergic receptor knockout male mice, testicular steroidogenic capacity is affected, Sertoli cell/Leydig cell communication is altered, and disruption of spermatogenesis was reported (80). Stojkov et al. (81) described that oral administration of an α_1_-adrenergic receptor blocker, mitigated stress induced disturbance of cAMP/cGMP signaling in testosterone-producing rat Leydig cells. An early study from Cohen et al. (82) reported that chronic exposure of the testicular vasculature to NE may cause spermatogenic dysfunction in subfertile men with varicocele. On the contrary, Nagao (83) proposed that Epi and NE may facilitate the *in vitro* viability of meiotic prophase spermatocytes in rats.

In hamster and human testicular macrophages, β_1_- and β_2_-adrenergic receptors were described and Epi and NE participated in the up-regulation of cyclooxygenase 2 (COX2) expression, prostaglandin production and the generation of local inflammatory processes (22). Laser microdissection followed by RT-PCR studies suggested that human testicular peritubular cells (HTPCs) of the wall of the seminiferous tubules express α_1B_-, α_1D_-, β_1_- and β_2_-adrenergic receptors. Phenylephrine, an α_1_-adrenergic receptor agonist, increased intracellular Ca^2+^-levels and induced inflammatory processes in cultured HTPCs (45). Therefore, elevation of catecholamines, for instance during stress, may also be able to promote inflammatory events by targeting these testicular cells.

It was further reported that stress increases testicular synthesis of HA in rats (84, 85). HA receptors H_1_ and H_2_ were found in germ cells, Leydig cells, Sertoli cells and peritubular cells of the human testis (60, 61). Human, mouse and rat Leydig cells also express H_4_ receptors (86–88).

HA regulates testosterone production in the MA-10 mouse Leydig tumor cell line, in primary culture of rat and wall lizard Leydig cells, and in testicular fragments of gonadally regressed hamsters kept in a short photoperiod (59, 87, 89). In histidine decarboxylase (HDC) knockout mice, the steroidogenic capacity of Leydig cells is significantly lower than in wild type mice (89). HA seems to exert a biphasic effect on testicular steroidogenesis in rodents (89). HA-induced stimulation of steroidogenesis seems primarily be mediated via H_2_ receptors and an increment of cAMP, HA-induced reduction of steroidogenesis may involve H_1_ receptors, stimulation of the PLC/IP3 pathway and increased nitric oxide synthase (NOS) activity (90). Via H_1_ and H_2_ receptors, HA also modulates the diameter of seminiferous tubules, testicular peritubular cells activity as well as sperm count and quality in different species (i.e. mouse, rat, bull, human) (61, 91–96). In testicular macrophages, HA inhibits phagocytosis and superoxide production, at least in the wall lizards (87).

In rodent Leydig cells, 5-HT_1A_, 5-HT_1B_ and 5-HT_2A_ receptors were found (43, 48, 97), and 5-HT and agonists of the 5-HT_1A_ and 5-HT_2A_ receptor subtypes inhibited cAMP and testosterone production, acting through the local CRH system and testicular α_1_- and β_1_-adrenergic receptors (43, 47, 97). 5-HT_3A_ receptors were reported in rat Sertoli cells, spermatogonia and spermatocytes, while 5-HT_1B_ and 5-HT_2A_ receptors were described in spermatogonia and sperm (48). Furthermore, 5-HT seems to be involved in the development of normal spermatogenesis and sperm quality in rats (98–100), as well as in the regulation of testicular blood flow (101).

MT_1_ and MT_2_ receptors were reported in mouse, rat, hamster and bovine Sertoli cells (55, 102, 103), where this indoleamine stimulates lactate dehydrogenase (LDH) expression and activity and, consequently, increases intracellular lactate production (55, 102). As recently described, daily oral melatonin supplementation positively regulates testicular expression of LDH (55) in men with idiopathic infertility. Melatonin also increased the levels of the glucose transporter 1 (GLUT1), glucose consumption and acetate production in rat Sertoli cells (102). Moreover, it upregulated pyruvate dehydrogenase E1 alpha subunit (PDHA1) phosphorylation in mouse and hamster Sertoli cells (55), inhibited the pyruvate dehydrogenase complex and increased the levels of pyruvate, which can be used to generate lactate via LDH activity (55).

Of note, melatonin abolishes the insulin-dependent increment of intracellular lactate and alanine, and the decrease of the lactate/alanine ratio in rat Sertoli cells controlling, in consequence, the intracellular redox state (102). In agreement with these results, Rossi et al. (55) reported that melatonin prevents the increase of lipid peroxidation and the decrease of the expression of antioxidant enzymes in hamster Sertoli cells.

Melatonin receptors are detectable in human and rodent testicular mast cells and macrophages (51). While melatonin inhibits proliferation and the expression of pro-inflammatory cytokines and COX2 in testicular macrophages, it increases the expression levels of enzymes of the anti-oxidant system and decreases the generation of reactive oxygen species (ROS) in testicular mast cells. Therefore, melatonin exerts anti-proliferative and anti-inflammatory effects on testicular macrophages and protective effects against oxidative stress on testicular mast cells (51).

In addition, MT_1_ receptors were identified in Leydig cells (49, 50). In MA-10 mouse Leydig tumor cells, as well as in primary cultures of rat and hamster Leydig cells, melatonin inhibited the production of sex steroid hormones (49, 104, 105). At least in the Syrian hamster, melatonin, through specific MT_1_ receptors of Leydig cells stimulated CRH production. CRH, via CRH-R1 receptors, activated tyrosine phosphatases leading to reduced phosphorylation of erk 44/42 and jnk 54/46, down-regulation of c-jun and c-fos, inhibition of transcription factors phosphorylation, decreased expression of StAR and finally inhibition of androgen production (43, 49, 50, 52). Melatonin can also regulate the expression of steroidogenic genes by binding to its nuclear receptors RORα in mammalian Leydig cells (106).

Circannual rhythms of circulating and testicular melatonin occur in response to changes of the daily photoperiod. This regulates steroidogenesis and the overall reproductive status of male Syrian hamsters, which are seasonal breeders (49, 107). In this species, the aging-related decrease of the testicular melatonin concentration was related to a diminished androgen production and an increment in inflammatory markers and indicators of oxidative stress (70, 108). Interestingly, daily i.p. injections of melatonin in old hamsters, as well as the transfer of aged animals from a long day to a short-day photoperiod, increased testicular melatonin levels and significantly improved the local inflammatory-oxidant status (70, 108). Particularly, hamster peritubular myoid cells of the tubular wall express MT_1_ and MT_2_ receptors, and melatonin regulates immune and inflammatory functions, as well as their contractile capacity (54).

In infertile men with unexplained azoospermia, lower testicular melatonin concentrations were found if testicular morphological abnormalities were present, together with increased macrophage numbers, elevated lipid peroxidation, expression of inflammation-related markers and antioxidant enzymes, as well as tubular wall collagen fibers disorganization and thickening (53). Consequently, testicular melatonin concentrations in biopsies of infertile men were negatively correlated with the gonadal levels of inflammatory markers (51, 53). In these patients, a daily oral supplementation with a 3 mg dose of melatonin, which is currently used to treat sleep disorders, not only increased the testicular levels of this indoleamine but also improved testicular inflammatory-oxidative status, as well as the tubular wall architecture (53). Therefore, melatonin appears to have a positive impact on testicular steroidogenesis and inflammatory-oxidative events.

## 4 Discussion

The studies mentioned clearly indicate that modulation of testicular functions can result upon interaction of biogenic amines with their target cells, Supplementary graphic summarizes the current knowledge about receptors and functions of biogenic amines in the different cell populations of the testis, However, this narrative also reveals that we only have a rudimentary knowledge of the actions of these signaling molecules in the testis. This has several reasons. For example, most of the investigations stem from earlier years and used traditional methods, including immunohistochemistry, RT-PCR or pharmacological tools (natural biogenic amines, agonists, antagonists). These approaches have obvious limitation as, for example, antibodies in some cases may lack specificity. Lack of specificity is also an issue for pharmacological agonist/antagonists. Furthermore, often isolated cells were examined and systemic studies were rarely performed. The fact that several species and different states of gonadal development or functionality were studied, may explain the sometimes contradictory results. Therefore, the currently limited knowledge about the role of biogenic amines in peripheral tissues calls for focused investigations in (systemic and cellular) models that resemble closely the human. They are required to answer the question, how important biogenic amines are for the male gonad especially in the human.

We believe that new studies trying to answer this question are worthwhile being performed, as biogenic amines, and drugs targeting their biosynthetic pathways and/or receptors are in daily clinical use or are being developed for a variety of conditions, yet their potential testicular consequences are not well known.

Looking at the widespread use of biogenic amines and/or their agonists/antagonists, in man, the importance of better insights becomes clear. In brief, a DA-precursor is used to treat Parkinson’s disease, DA-agonists are used in human prolactinomas, acromegaly, and in type 2 diabetes (109). Epi, NE and agonists of adrenergic receptors are used as vasopressors in acute hypotensive states, as vasoconstrictors to reduce bleeding, and as nasal decongestants, but also in the treatment of anaphylaxis and bronchospasms. Adrenergic receptors antagonists are employed to treat hypertension, congestive heart failure, and some cardiovascular diseases. Inhibitors of catecholamine reuptake are part of several psychiatric treatments and used against musculoskeletal pain (110). 5-HT reuptake inhibitors are prescribed to treat depression (111, 112). Melatonin is prescribed to improve sleep in men suffering from insomnia and jet lag (113). As a consequence of its strong antioxidant action, the therapeutic potential of melatonin was suggested to be extended to Parkinson’s disease, cancer and male infertility (53, 114, 115). Finally, anti-HA medications and mast cell stabilizers are frequently used in allergic diseases (116).

Of note, there is vast body of evidence showing that catecholamines interact with cells of the immune system (117–120). Immune cells and innervation density and pattern in the male gonad change during development and infertility (20–22, 31, 32). Yet, the clinical implications of the interplay of adrenergic compounds and local immune cells of the testis, have not yet been investigated.

Furthermore, adrenergic nerves are important players in the aging of hematopoietic stem cell niche in bone marrow of mice (121). The testis also harbors a stem cell niche, yet the possibility of an influence of testicular nerves and biogenic amines on the spermatogonial stem cell niche is not known, neither in young adult nor in middle-aged adult and aged men. With exception of melatonin, for which current findings in rodents indicate a potential beneficial role on the steroidogenic activity and the local inflammatory-oxidative status in aged testes (70, 108, 122), biogenic amines and testicular aging represent almost unexplored field of research.

We believe that the time for new studies of the old topic “biogenic amines and their roles in the testis”, yet with a focus on the clinical, i.e. human-specific aspects, may have come. Modern techniques will provide unprecedented novel insights and may identify adequate models. In particular, (spatial) multiomics approaches (123, 124) will be instrumental, i.e. the integration of molecular (DNA, RNA, protein) data and lineage data, possibly with three-dimensional spatial resolution (or even four-dimensional, temporal resolution). Indeed, more and more single cell sequencing studies are already being published (125, 126) and together with other approaches (127, 128), they eventually will provide a clear and precise picture of the sources of biogenic amines, and their testicular receptors, as well as their changes during development, aging and diseases.

## Author contributions

MF: Conceptualization, Writing – original draft, Writing – review & editing. AM: Conceptualization, Writing – original draft, Writing – review & editing.
