# Supplementary figures and images for: Biogenic amines in the testis: sources, receptors and actions
Source: Front Endocrinol (Lausanne). 2024 Jun 20;15:1392917. doi: 10.3389/fendo.2024.1392917 (PMC11222591; doi:10.3389/fendo.2024.1392917)

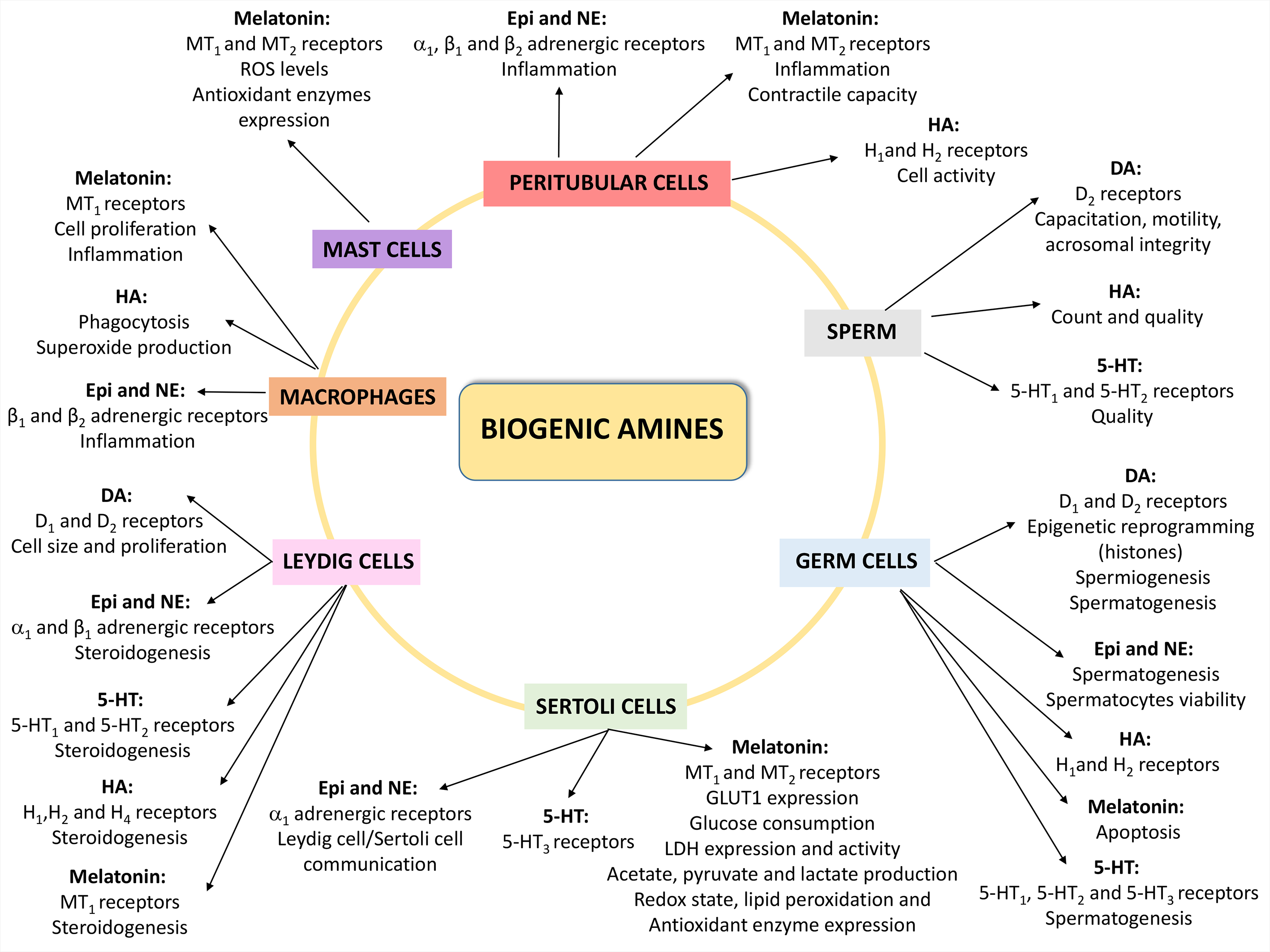

Supplement: Supplementary file 1 [file Image_1.tif]
